# Factors affecting the accuracy of urine-based biomarkers of BSE

**DOI:** 10.1186/1477-5956-9-6

**Published:** 2011-02-07

**Authors:** Margot Plews, Lise Lamoureux, Sharon LR Simon, Catherine Graham, Viola Ruddat, Stefanie Czub, J David Knox

**Affiliations:** 1 Prion Diseases Program, Public Health Agency of Canada, Winnipeg, R3E 3P6, Canada; 2 Animal Diseases Research Institute, Canadian Food Inspection Agency, Lethbridge, T1J 3Z4, Canada; 3 GE Healthcare, San Francisco, CA 94110, USA; 4 Dept. of Medical Microbiology, University of Manitoba, Winnipeg, MB R3T 2N2, Canada

## Abstract

**Background:**

Transmissible spongiform encephalopathy diseases are untreatable, uniformly fatal degenerative syndromes of the central nervous system that can be transmitted both within as well as between species. The bovine spongiform encephalopathy (BSE) epidemic and the emergence of a new human variant of Creutzfeldt-Jakob disease (vCJD), have profoundly influenced beef production processes as well as blood donation and surgical procedures. Simple, robust and cost effective diagnostic screening and surveillance tools are needed for both the preclinical and clinical stages of TSE disease in order to minimize both the economic costs and zoonotic risk of BSE and to further reduce the risk of secondary vCJD.

**Objective:**

Urine is well suited as the matrix for an ante-mortem test for TSE diseases because it would permit non-invasive and repeated sampling. In this study urine samples collected from BSE infected and age matched control cattle were screened for the presence of individual proteins that exhibited disease specific changes in abundance in response to BSE infection that might form the basis of such an ante-mortem test.

**Results:**

Two-dimensional differential gel electrophoresis (2D-DIGE) was used to identify proteins exhibiting differential abundance in two sets of cattle. The known set consisted of BSE infected steers and age matched controls throughout the course of the disease. The blinded unknown set was composed of BSE infected and control samples of both genders, a wide range of ages and two different breeds. Multivariate analyses of individual protein abundance data generated classifiers comprised of the proteins best able to discriminate between the samples based on disease state, breed, age and gender.

**Conclusion:**

Despite the presence of confounding factors, the disease specific changes in abundance exhibited by a panel of urine proteins permitted the creation of classifiers able to discriminate between control and infected cattle with a high degree of accuracy.

## Introduction

Transmissible spongiform encephalopathy (TSE) diseases are untreatable, uniformly fatal degenerative syndromes of the central nervous system that can be transmitted both within as well as between species. Bovine spongiform encephalopathy (BSE) of cattle, chronic wasting disease (CWD) of cervids, scrapie of sheep and a new variant of Creutzfeldt-Jakob disease (vCJD) of humans, thought to be acquired through dietary exposure to BSE infect beef as well as iatrogenically, are among the most notable TSE diseases[1,2].

In response to the association of dietary exposure to BSE infected beef and the 1996 emergence of vCJD a variety of risk reduction measures were put in place in the U.K. Specified risk material (SRM), consisting of tissues known to harbour high levels of infectivity such as the brain and spinal cord were removed from the food chain and between 1996-2005 all animals of ≥30 months of age were also excluded. The introduction of rapid tests for BSE in 2005 allowed cattle ≥30 months of age to enter the food chain provided they tested negative for BSE. Subsequently, in 2008 the countries of the EU raised the BSE testing age to 48 months[3]. These measures are presumed able to prevent dietary exposure to contaminated tissue.

A hallmark of TSE diseases is an accumulation of misfolded isoforms of a host-encoded protein, PrP, or prion protein. The BSE rapid tests rely upon the detection of the disease associated host-encoded prion protein, PrP^d^, in brain tissue post-mortem and are consequently unsuitable for assessing the health of breeding stock. Such tests would also be unsuitable for performing CJD surveillance of prospective blood donors or people undergoing surgical procedures. The risk of secondary transmission of vCJD has been demonstrated by four cases of vCJD observed in persons that received transfusions of packed red blood cells from asymptomatic donors who subsequently died from vCJD[1]. It has also been recently recognized that heterozygotes might be similarly susceptible to BSE infection as those homozygous methionine at the polymorphic codon of the prion gene. This would heighten the risk of secondary vCJD transmission because heterozygotes represent a larger proportion of the population exposed to BSE tainted beef and these individuals remain asymptomatic and potentially infectious for extended periods of time [4,5]. Thus, there is continued demand for an ante-mortem TSE test based on a matrix or body fluid that would permit repeated sampling.

Diagnostic screening and surveillance tools need to be simple, robust and cost effective. Ideally, such a test would be comprised of one protein. However, in reality finding a panel of markers is more likely as well as sufficient. The use of the disease related isoform, PrP^d^, as a pre-clinical or general marker for surveillance has been limited, due to the fact that it is present in extremely small amounts in accessible tissues and bodily fluids. Specific detection of these small amounts of the PrP^d ^conformer are further exacerbated by the presence of large excesses of endogenous PrP. Efforts to surmount these problems have relied on the partial protease resistance of PrP^d ^coupled with signal amplification by the protein misfolding cyclic amplification assay (PMCA)[6-8]. Despite early optimism the demonstration that infectivity is associated with protease sensitive forms of PrP^d ^and that PMCA is able to produce PrP^d ^*de novo *means that such techniques are currently unable to form the basis of an ante-mortem test[9,10]. To circumvent these problems an alternative, but as yet unproven strategy has been to identify differentially abundant host proteins characteristic of TSE infection to act as surrogate markers of disease[11-13].

Previously, we used 2D-DIGE to perform an unbiased screen of the urinary proteome of a small cohort of Fleckvieh-Simmental cows. The results demonstrated that the relative abundance of a particular isoform of the glycoprotein clusterin was able to accurately discriminate between BSE infected and age matched control cattle as early as 8 months post infection[13,14]. The animals of this cohort were all females of the same breed, inoculated at 4 months of age, and raised in the same location. The uniformity of this data set was well suited for the identification of disease specific changes in the abundance of particular proteins. In contrast, more stringent criteria must be applied to biomarker identification as biomarkers are required to exhibit a reliable response in a heterogeneous population. To start to address these issues this study describes the analysis of both a known set and an associated blinded unknown set that contained BSE infected cattle and control cattle of different breeds, genders and ages. All three traits are demonstrated to significantly affect the urinary proteome. Nonetheless, despite these confounding factors disease specific changes in the individual abundance of a panel of urinary proteins were able to discriminate between control and infected cattle with a high degree of accuracy.

## Materials and methods

### Urine

Frozen urine samples (20 mL) were received from the Veterinary Laboratory Agency (VLA, Weybridge, UK) and stored at -80°C. The known set consisted of 8 neutered males; 4 infected with a 100 g oral dose of BSE at 4-5 months of age and 4 age matched controls. The breed of the steers was either Friesian or crossbred Friesian-Holstein. Urine samples were collected longitudinally at 5 to 6 month intervals for the first 41 months of the disease. The blinded unknown set consisted of 19 samples representing 19 individual animals of indeterminate disease status. Animal use for scientific purposes was reviewed by the VLA Ethics Committee. The VLA is bound by the Animals (scientific procedures) Act 1986 which is administered by the Home Office. All samples were processed as described previously [13].

Urine samples were thawed overnight at 4°C. Insoluble particles were removed by a pre-spin, 4°C at 415 g for 5 minutes. The soluble fraction was concentrated with a 5 K MWCO Centricon Plus-70 centrifugal filter unit (Millipore) in a swinging bucket rotor at 4°C and 3400 g for approximately 20 minutes or until volumes reached less than 4 mL. The urine was further concentrated with an Amicon 4 ml 5 K MWCO centrifugal filter unit (Millipore) at 4°C and 7000 g until volumes reached 200 μl.

The concentrated urine samples were purified using a 2D Clean Up Kit (GE Healthcare) according to the manufacturer's recommendations. The resulting protein pellets were resuspended in 100 μL of DIGE labelling Buffer (0.03 M Tris, 8 M Urea, 2 M Thiourea, 2% Chaps, pH 8.5). Samples were adjusted to pH8.5 with the addition of 1-5 μl of 0.05 M NaOH. The concentration of each sample was determined using a 2D Quant Kit (GE Healthcare) according to the manufacturer's recommendations. An internal standard was created by combining 100 μg of each sample.

### 2D Gel Electrophoresis

CyDye™ (GE Healthcare) minimal labelling was performed as per the manufacturer's recommendations (400 pmol: 50 μg) with the Cy2 label reserved for the pooled standard. The control and infected samples were labelled in a randomized manner with either Cy3 or Cy5. An equal volume of 2 × Rehydration Buffer (0.03 M Tris, 8 M Urea, 2 M Thiourea, 2% Chaps, pH 8.5, 4 mg/ml Dithiothreitol (DTT), 1% IPG buffer pH 4-7) was added to a mixture comprised of 30 μg each of the labelled Pooled, Control and Infected samples. After a 10 minute incubation on ice approximately 400 μl of 1 × Rehydration Buffer (0.03 M Tris, 8 M Urea, 2 M Thiourea, 2% Chaps, pH 8.5, 2 mg/ml DTT, 0.5% IPG buffer pH 4-7) and 5 μl of 1% Bromophenol blue (10 mM TrisCl pH 8.5) were added to bring the volume up to a total of 450 μl. The labelled samples were loaded onto a reswelling tray and overlaid with a 24 cm Immobiline DryStrip pH 4-7 (GE Healthcare) and DryStrip Cover Fluid for rehydration overnight at room temperature. The strip was transferred to a Manifold filled with 108 mL of DryStrip Cover Fluid and placed on an Ettan IPGphor3 focusing apparatus that was programmed as follows: Step 30 V 8 hrs, Step 500 V 1 hr, Step 1000 V 1 hr, Grad 10000 V 3 hrs and Step 10000 V 3 hrs. A final focusing program: Grad 10000 V 0:20 hr and Step 10000 V until the volt hours reached a total of 55000 was added, as required.

Completed isoelectric focusing (IEF) strips were stored at -80°C until the second dimension was started. IEF strips were prepared for second dimension gels by incubating in two different Equilibration Buffer solutions (50 mM Tris-Cl pH8.8, 6 M Urea, 30% Glycerol, 2% SDS, 0.2% Bromophenol Blue, supplemented with either 65 mM DTT - 1st incubation or 135 mM Iodoacetamide - 2^nd ^incubation) for 15 minutes each with gentle rocking. The equilibrated IEF strips were placed on 15-20% gradient gels between low fluorescent glass plates (NextGen Sciences). After sealing in place with a 1% agarose solution, the gels were placed in the Ettan DALT6 unit (GE Healthcare) and run at 2 W overnight and then at 100 W until a total of 3100 Vhr was reached.

### Gel image acquisition

Gels were scanned with a Typhoon 9410 variable mode imager. The known set consisted of 4 infected and 4 control biological replicates at each of the 7 time points. An exception was infected steer #31 that was culled prior to 41 months post infection (mpi, Table 1). In total 28 gels, each comprised of the internal standard and one or two biological samples were run to produce 55 gel images representing 55 biological samples with matching images of the internal standard. The blinded unknown set (Table 2) consisted of 20 samples. Unknown sample #2 was not analyzed as a suitable gel image was not obtained. The remaining 19 individual animals of indeterminate disease status were run on ten gels that contained the same Cy-2-labelled internal standard as used for the known set above.

**Table 1:** Samples of the Known Set.

			MPI							Age of animal at terminal collection
Animal ID	Gender	Breed	6	11	17	23	29	35	41	
ctl 3	male	Friesian/Holstein	√	√	√	√	√	√	√	4 years
ctl 8	male	Friesian/Holstein	√	√	√	√	√	√	√	5 years, 1 month
ctl 11	male	Friesian	√	√	√	√	√	√	√	4 years, 1 month
ctl 1822	male	Friesian/Holstein	√	√	√	√	√	√	√	4 years, 10 months

inf 5	male	Friesian	√	√	√	√	√	√	√	3 years, 11 months
inf 9	male	Friesian	√	√	√	√	√	√	√	3 years, 11 months
inf 22	male	Friesian	√	√	√	√	√	√	√	4 years, 2 months
inf 31	male	Friesian/Holstein	√	√	√	√	√	√	X	3 years, 5 months

The known set consisted of eight neutered males. Four were orally infected with 100 g of BSE infected brain homogenate. Urine samples were collected at 5-6 month intervals post infection from both the infected and age matched control steers. The breed of the steers is asymmetrically divided between the two groups. The infected group consists of three Friesians and one crossbred Friesian-Holstein. Conversely the control group consists of the crossbred Friesian-Holsteins and one Friesian.

**Table 2:** Samples of the Unknown Set.

Unknown Sample #	Gender	Breed	Age	Disease	Classification
1	male	Friesian/Holstein	10 years, 4 months	negative	negative

3	female	Friesian	4 years, 9 months	negative	negative

4	female	Friesian/Holstein	10 years, 7 months	positive	positive

5	female	Friesian	4 years, 8 months	positive	positive

6	female	Friesian	4 years, 9 months	negative	negative

7	male	Friesian/Holstein	10 years, 7 months	negative	positive

8	female	Friesian	8 years, 1 month	positive	positive

9	female	Friesian/Holstein	5 years, 0 months	positive	negative

10	female	Friesian	4 years, 9 months	negative	negative

11	female	Friesian	5 years, 5 months	negative	positive

12	female	Friesian	10 years, 4 months	positive	positive

13	female	Friesian	9 years, 4 months	positive	positive

14	male	Friesian/Holstein	10 years, 4 months	negative	negative

15	female	Friesian	10 years, 4 months	positive	positive

16	male	Friesian/Holstein	10 years, 4 months	negative	positive

17	female	Friesian	5 years, 5 months	negative	positive

18	female	Friesian/Holstein	5 years, 0 months	positive	positive

19	female	Friesian	4 years, 8 months	positive	positive

20	female	Friesian	5 years, 5 months	negative	negative

The gender, breed and age of the animal from which the samples were obtained are given in columns two, three, and four respectively. In column five the true disease state of the animals is given and in column 6 it is indicated how the samples were classified by the known set generated classifier. A suitable gel image of sample #2 was not obtained.

### Analysis

**Figure 1:**
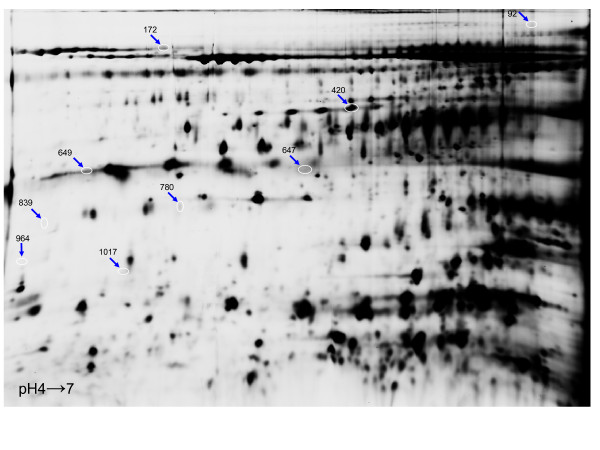
**Representative Cy2-labelled internal standard proteome gel image illustrating proteins resolved in the pH4-7range**. The gel image as loaded into the DIA module prior to spot detection. The blue arrows indicate the nine spot features of the known set that comprised the classifier used to classify the unknown set.

The acquired gel images were analyzed using the DeCyder™ Differential In-gel Analysis (DIA) and Biological Variation Analysis (BVA) software modules (version 7.0). Manual landmarking of spot features across all internal standard images was performed in order to improve the accuracy of the gel-to-gel matching process. This resulted in the detection, quantification, and matching on average 1143 master spot features/gel across the 38 gels (Figure 1). Classifiers were generated by the algorithms of the DeCyder™ Extended Data Analysis Software (EDA) module using the default settings for cross validation. Cross validation involves partitioning the data into complementary subsets, performing the analysis on one subset and validating the analysis on the other subset. To reduce variability, multiple rounds of cross validation are performed using different partitions and the results are averaged over the rounds. Two parameters indicating the quality of the proteins comprised in the classifiers are calculated by the DeCyder™ software; appearance (A), the number of classifiers that have selected this protein, and rank (R), the mean value of the different classifiers ranking of the proteins. The master spot features used to generate the classifiers and PCA plots as well as their rank and appearance values are provided in Additional file 1, Table S1.

## Results and Discussion

### BSE biomarkers

**Figure 2:**
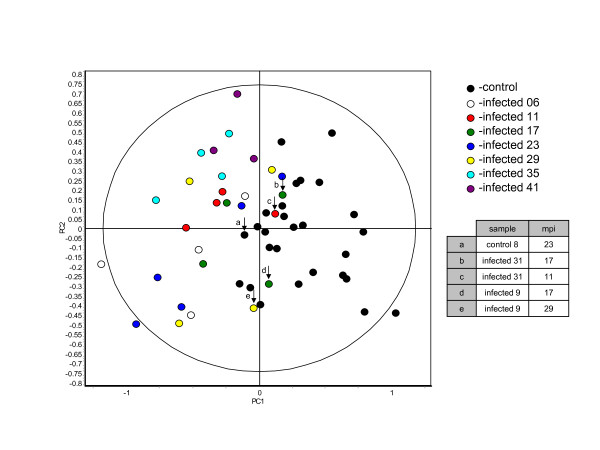
**Principle component analysis of the 55 biological samples of the known set using the nine spots features best able to discriminate between control and infected samples**. Five samples are misclassified using this classifier producing one false positive and four false negatives. The arrows indicate the misclassified samples and the table provides their identity. The only similarity between the misclassified samples is that they are relatively early in the disease course. (PC1 = 48.8, PC2 = 20.6)

Multivariate analyses of the BVA protein expression data derived from the known set were performed using the EDA software module. The gel images of the known set were grouped according to disease state and months post infection (mpi). Grouping the 55 gel images of the known set according to disease state and mpi generated 14 groups each representing either the four infected or four control animals at particular time points. The data were filtered so that only the 103 spot features present in ≥80% of the gels and exhibiting statistically significant (ANOVA p ≤ 0.01) changes in abundance were considered in the following analyses. The 103 spot features were searched and evaluated by the forward selection (FS) and regularized discriminant analysis (RDA) algorithms to identify the subset of spot features best able to discriminate between control and infected samples. Nine selected spot features were used to create an RDA generated classifier that classified the control and infected samples of the known set with 90.7 ± 7.1% accuracy. A PCA based on the classifiers as features was carried out, illustrating a demarcation between the control and infected animals (Figure 2). The correct classification of the known set samples obtained from animals early in the disease course (6 mpi) demonstrated that changes in the abundance of some proteins occurs rapidly after BSE infection via the oral route (Figure 2). This confirmed the results observed previously with Fleckvieh-Simmental cows[13].

**Figure 3:**
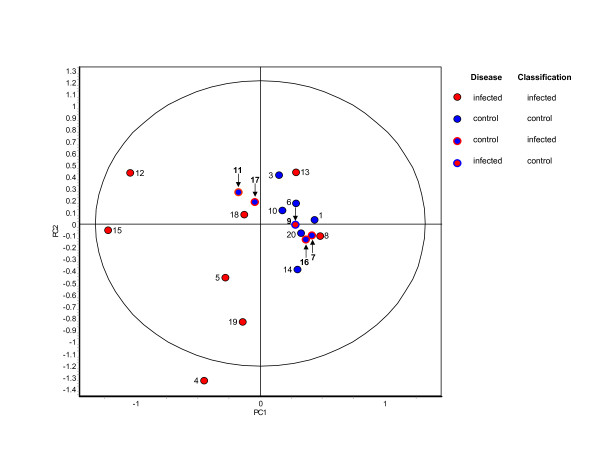
**Principle component analysis of the unknown set using the classifier generated by the known set**. The number adjacent to each spot identifies the unknown set sample it represents. The colour of the centre of each circle identifies its true disease state (blue = BSE negative, red = BSE positive). The positions of the misclassified samples (7, 9, 11, 16, 17) are indicated by arrows. The perimeter colour indicates how these samples were classified by the known set classifier as opposed to their true identity indicated by the colour of the interior of the circles. When applied to the unknown set the known set classifier generates four false positives and one false negative. (PC1 = 40.5, PC2 = 22.5).

The high degree of the calculated accuracy indicated that the disease status of the animals was a major influence affecting the differential abundance of individual proteins in urine. However, a true measure of the performance of the classifier requires that it be applied to an independent data set not involved in the determination of the classifier parameters. When applied to the 19 samples in the independent unknown data set the classifier generated by the known set was able to discriminate between control and infected samples with 74% accuracy. The classification resulted in 4 false positives and 1 false negative. Thus in the context of detecting infected cattle the classifier performed well in comparison to the detection of 14-3-3 proteins in cerebral spinal fluid as a marker for sporadic CJD patients[15,16]. Principle component analysis was carried out on the 19 samples of the unknown data set using the nine features that comprised the classifier to visually illustrate classification of the spot maps (Figure 3).

### Confounding Factors

An assumption built into the assessment of the true accuracy of the classifier was that both the known and unknown sets were representative of the same 'cow population'. Inspection of the details of the cattle from the known and unknown sets revealed that in fact the cattle populating the two data sets were dramatically different. One factor well known to influence urine composition is gender[17]. Gender differences surely played a role as the known set was composed entirely of neutered males while 15 of the 19 animals of the blinded unknown set were females (Table 2). Age of the animals has also been shown to influence transcriptional profiles and the urine proteome[13,18]. All the animals of the known set were under 5.5 years of age. In contrast, 9 of the 19 animals of the unknown set were 8 years of age or older (Table 2). Tabulating the known information about the source of the urine samples clearly illustrates that the samples of the known set used to create the classifier were not representative of the 'cow population' of the blinded unknown set (Table 3).

**Table 3:** Compare and Contrast the Samples of the Known and Unknown Sets.

Unknown Set					
**Age of Animals When Samples Obtained**	**BSE Status**	**male**		**female**	
		
		**F**	**HxF**	**F**	**HxF**

≥ 8 years old	Positive	0	0	4	1
	Negative	0	4	0	0

≤ 6 years old	Positive	0	0	2	2
	Negative	0	0	6	0

**Known Set**					

≥ 8 years old	Positive	0	0	0	0
	Negative	0	0	0	0

≤ 6 years old	Positive	3	1	0	0
	Negative	1	3	0	0

The animals producing the samples of the known set were all relatively young steers. In contrast 15 of the samples of the test set were produced by females and five of these were much older in age than the samples of the known set. The unknown set also included four steers, but none of these were of the same age range as the steers of the known set. Note that the known set animals are located in the bottom left quadrant of the table that is unpopulated by animals of the unknown set.

Another variable came to light once all the parameters of the unknown set were known. When choosing the known set we had purposely selected a longitudinal series of samples in order to determine how early in the disease course we would be able to identify BSE positive animals. In contrast, the infected urine samples of the unknown set were all obtained from naturally infected animals exhibiting clinical signs indicative of BSE and subsequently definitively diagnosed as BSE positive by post mortem analysis.

### Creation of a Merged Sample Set

The difference in disease stage between the two data sets can be lessened by considering just the last sample collected from each animal of the known set. At this time point (35 or 41 mpi) the infected animals of the known set were all showing clinical symptoms of BSE infection making them comparable to the infected samples of the unknown set. All control animals in both the known and unknown sets were non BSE exposed and in good physical shape. A merged set containing the last sample collected from each of the 8 animals of the known set with the 19 individual samples of the unknown set was formed to determine the contributions of breed, age and gender to the protein profile of urine (Additional file 2, Table S2).

### Breed

**Figure 4:**
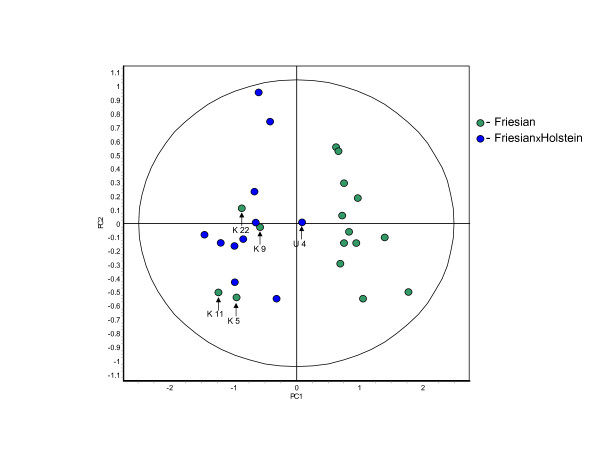
**Principle component analysis of samples grouped according to breed**. To lessen the effects of differences due to disease stage the 27 samples of the merged set consist of the eight terminal samples of the known set and all the samples of the unknown set. When grouped according to breed 122 proteins were present on 80% of the gels and showed significant differential abundance (ANOVA p ≤ 0.01). The 122 proteins were analyzed using the FS and RDA algorithms to identify those proteins best able to discriminate between the two breeds of cattle. RDA was used to generate a classifier out of the 12 selected proteins that was able to correctly classify the samples with 82.5 ± 12.6% accuracy. The misclassified samples are identified by arrows and the sample number to which they correspond. (K = known set, U = unknown set, PC1 = 70.2, PC2 = 12.3).

Prior analyses of the known set, where age and gender were not factors, clearly demonstrated that the different breeds differentially influenced the urine protein profile (Additional file 3, Figure S1). A new classifier was created based on different breeds. When applied to the merged set it too demonstrated that breed influenced the abundance of a panel of proteins to a significant degree (82.5 ± 12.6%). The resulting PCA based on these features shows the misclassified Friesians as part of the cluster of FriesianXHolsteins and the one misclassified FriesanXHolstein was closer to the Friesian cluster (Figure 4). Five samples of the merged set were misclassified. Interestingly, 4 of the misclassified samples were Friesian steers of the known set that were correctly classified with respect to breed when the known set was analyzed in isolation. This indicated that factors in addition to disease state and breed were having a marked influence on the protein composition of urine. The other misclassified sample was unknown set #4. The crossbreed Friesian-Holstein infected cow that produced this sample was the oldest animal in the merged set suggesting that age might also play a role.

### Age

**Figure 5:**
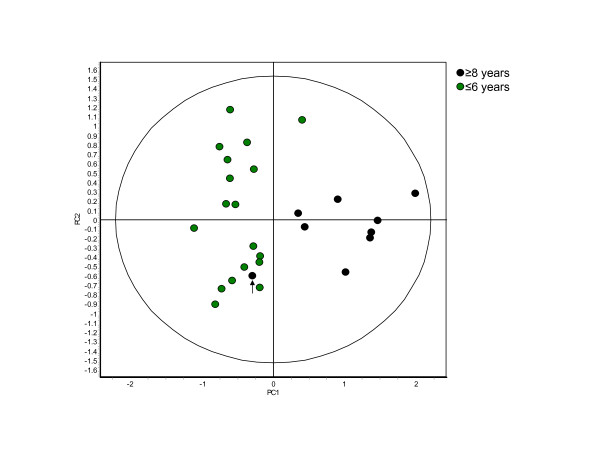
**Principle component analysis of the samples grouped according to age**. The 27 samples of the merged set were divided into two groups. One containing the 9 animals over 8 years of age and the other containing the 18 animals under 6 years of age. The 44 proteins present in 100% of the gels and exhibiting significant differential abundance (ANOVA p ≤ 0.01) were analyzed using the partial least squares search and RDA algorithms to identify those proteins best able to differentiate between the samples based on the age of the animal that produced them. RDA was used to generate a classifier out of the 5 selected proteins that was able to correctly classify the samples with 95.0 ± 11.2% accuracy. The misclassified sample, unknown set sample 13, is indicated by an arrow. (PC1 = 56.2, PC2 = 27.0).

The age of the animals producing the samples of the merged set ranged from 3.4 to 10.6 years of age. The absence of animals between the ages of 5.4 and 8.1 years of age provided a clear demarcation between the 9 samples to be considered 'old' from the 19 considered to be 'young' (Table 3). When grouped as old and young with respect to the animals that produced them a classifier created to discriminate age correctly classified the samples with 95.0 ± 11.2% accuracy (Figure 5). PCA, using the 5 features of this classifier visually illustrates the misclassified Friesian cow of age 9.3 in midst of the younger cows.

### Gender

**Figure 6:**
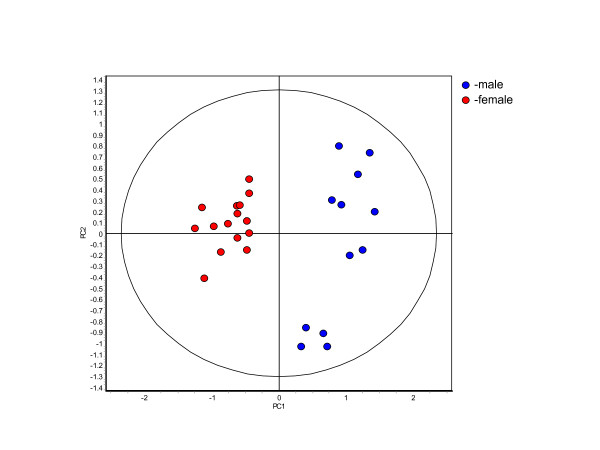
**Principle component analysis of the samples grouped according to gender**. The 27 samples of the merged set were divided into two groups. One group contained the 12 steers and the other contained the 15 cows. The 185 proteins present in 100% of the gels and exhibiting significant differential abundance (ANOVA p ≤ 0.01) were analyzed using the PLSS and RDA algorithms to identify those proteins best able to differentiate between the samples based on the gender of the animal that produced them. RDA was used to generate a classifier out of the 4 selected proteins that correctly classified all the samples with respect to gender. (PC1 = 66.8, PC2 = 20.7).

When grouped according to the gender of the animal that produced them the samples of the merged set are broken into a group of 15 samples produced by females and a group of 12 samples produced by neutered males. Discriminant analysis of gender resulted in a classifier that could correctly classify gender with 100% accuracy. PCA based on the four features of this classifier resulted in two completely separated clusters illustrating the successful classification based on this classifier (Figure 6). The PCA plot was also a clear example that despite being focused on gender the influence of other factors are not removed. The four male samples clustered together in the lower right hand quadrant of the PCA plot are all BSE negative, Friesian-Holstein crossbreeds and over 10 years of age.

### Disease Status

**Figure 7:**
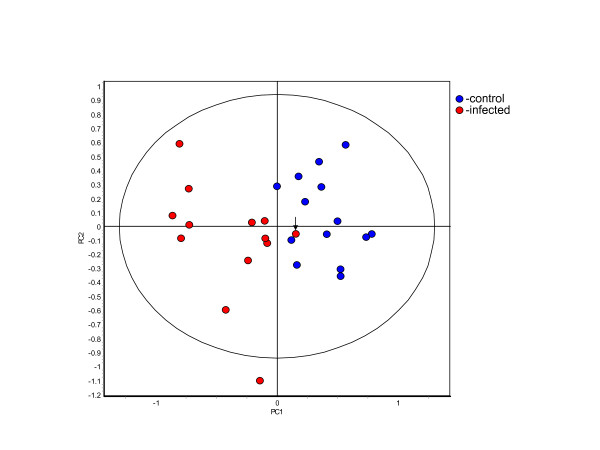
**Principle component analysis of the samples grouped according to disease state**. The 27 samples of the merged set were divided into two groups. One group contained the 14 BSE negative samples and the other group contained the 13 BSE positive samples. The 42 proteins present in 80% of the gels and exhibiting significant differential abundance (ANOVA p ≤ 0.01) were analyzed using the PLSS and RDA algorithms to identify those proteins best able to differentiate between the samples base upon the disease state of the animal that produced them. RDA was used to generate a classifier out of the 10 selected proteins that were able to correctly classify the samples with 96.7 ± 7.5% accuracy. The one misclassified sample, known set sample 22, is identified by an arrow. (PC1 = 48.4, PC2 = 26.0).

Ultimately, it was of interest to determine how accurate a classifier based on disease induced changes in the abundance of particular proteins could be created in samples where breed, age and gender significantly affected the protein profile of the urine samples. When the samples of the merged set were grouped based upon their disease state a group of 13 samples produced by BSE positive animals and another group of 15 samples produced by BSE negative animals were generated. When grouped in this fashion a new clinical stage classifier was able to sort the control and infected samples of the merged set with 96.7 ± 7.5% accuracy. Applying PCA to this set of 10 features in the classifier, resulted in only one infected animal as part of the control cluster (Figure 7).

## Conclusions

Our earlier observation, that BSE induced changes in the protein profile of urine occur shortly after oral infection, was confirmed. This result suggests that the diagnosis of acquired TSE diseases may be possible at a time prior to the accumulation of irreversible damage when future interventions may be most effective. Discrepancies between the known and unknown sets precluded a true assessment of the performance of the classifier produced by the known set. Nonetheless, by creating a merged set combining the last sample obtained from each animal of the known set with the samples of the unknown set we were able to gauge the effects of breed, age, and gender as well as disease state. The results demonstrated that, at the clinical stage of the disease, factors such as breed, age and gender significantly affected the protein profile of urine. Despite these confounding factors disease specific changes in the abundance of a panel of proteins, able to discriminate between control and clinical stage infected cattle, can be observed in this complex data set. A true measurement of the performance of the classifier generated by the merged set will require the future testing of naive sample sets. Once validated the proteins of the classifier would be individually identified.

## Abbreviations

BVA: Biological Variation Analysis; BSE: Bovine Spongiform Encephalopathy; CWD: Chronic wasting disease; CJD: Creutzfeldt-Jakob disease; DIA: Differential In-gel Analysis; EDA: Extended Data Analysis; FS: Forward selection; mpi: months post infection; PCA: principle component analysis; PrP: prion protein; PMCA: protein misfolding cyclic amplification assay; RDA: regularized discriminant analysis; TSE: Transmissible spongiform encephalopathy; vCJD: variant Creutzfeldt-Jakob disease

## Competing interests

The authors declare that they have no competing interests.

## Authors' contributions

MP, SLRS and LL participated in experimental design, data acquisition and critical revisions the manuscript. CG and SC contributed to the conception of the project and critically revised the manuscript. VR contributed to experimental design and critically revised the manuscript. JDK conceived the project, analysed and interpreted the data and drafted the manuscript. All authors read and approved the final manuscript.

## Supplementary Material

Additional file 1 **Table S1 Master Spot Features Used as Classifiers**. The spot features used to generate each of the classifiers discussed and the corresponding figures are listed. The appearance (A) and rank (R) of each protein are parameters indicating the quality of the protein relative to the other proteins included in each classifier. Click here for file

Additional file 2 **Table S2 The Merged Set**. The last urine sample obtained from each of the 8 animals of the known set was added to the 19 samples of the unknown set to create a set referred to as the merged set. The gender, breed, age, and disease status of these 27 animals are given in columns 2-5 respectively. Click here for file

Additional file 3 **Figure S1 The Effect of Breed on the Urine Protein Profiles of the Known Set**. The 55 samples of the known set were divided into two groups based on breed. The 126 proteins present in 80% of the gels and exhibiting significant differential abundance (ANOVA p ≤ 0.01) were analyzed using the FS and K-Nearest Neighbours algorithms to identify those proteins best able to differentiate between the samples based upon the breed of animal that produced them. RDA was used to generate a classifier out of 8 selected proteins that correctly classified the samples with 94.7 ± 8.1% accuracy with respect to breed. The three misclassified samples are indicated by arrows. (PC1 = 43.2, PC2 = 24.1). Click here for file
