# Supplementary figures and images for: Factors affecting the accuracy of urine-based biomarkers of BSE
Source: Proteome Sci. 2011 Feb 7;9:6. doi: 10.1186/1477-5956-9-6 (PMC3045280; doi:10.1186/1477-5956-9-6)

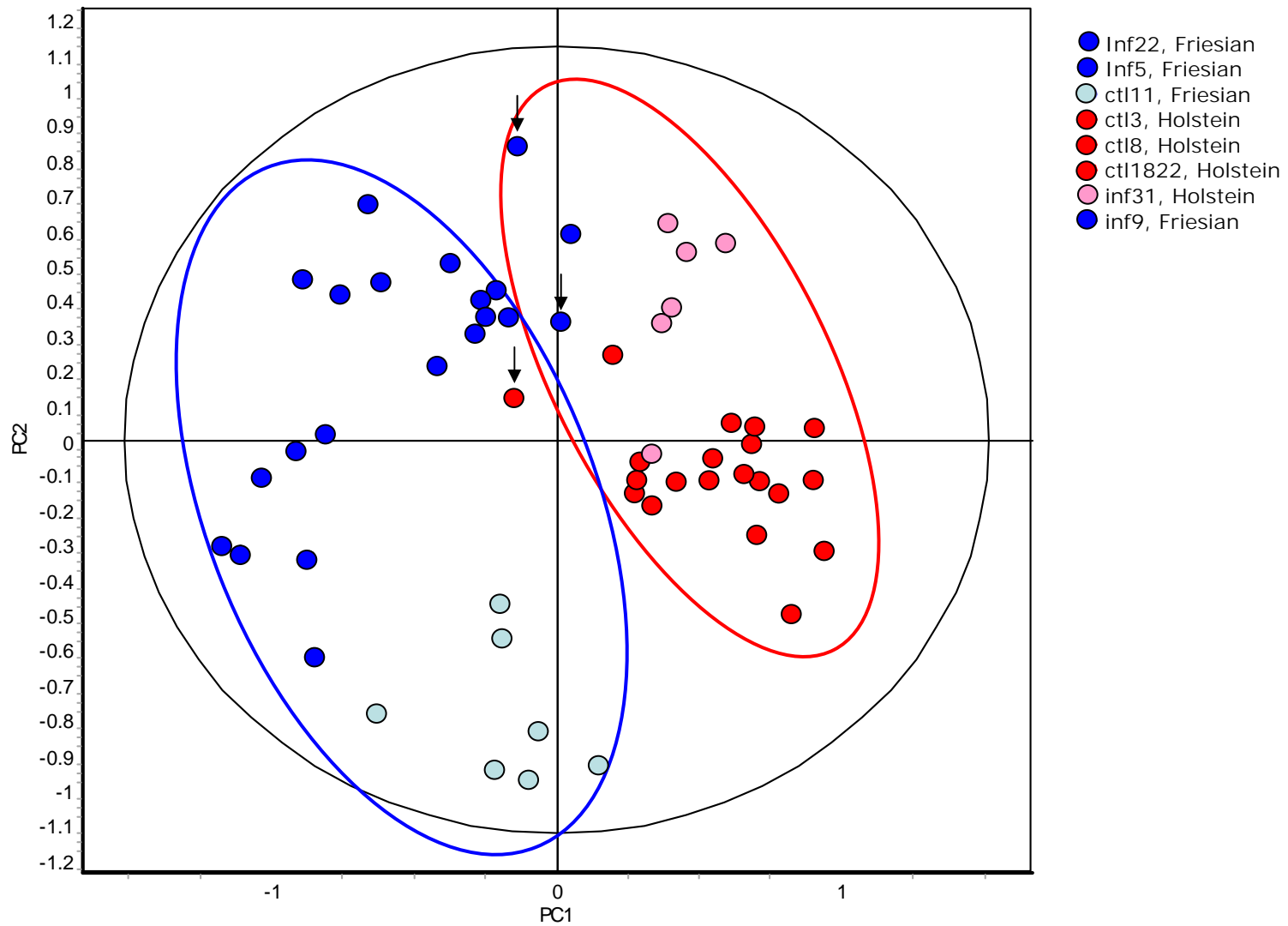

Supplement: Additional file 3 — Figure S1 The Effect of Breed on the Urine Protein Profiles of the Known Set. The 55 samples of the known set were divided into two groups based on breed. The 126 proteins present in 80% of the gels and exhibiting significant differential abundance (ANOVA p ≤ 0.01) were analyzed using the FS and K-Nearest Neighbours algorithms to identify those proteins best able to differentiate between the samples based upon the breed of animal that produced them. RDA was used to generate a classifier out of 8 selected proteins that correctly classified the samples with 94.7 ± 8.1% accuracy with respect to breed. The three misclassified samples are indicated by arrows. (PC1 = 43.2, PC2 = 24.1). [file 1477-5956-9-6-S3.PDF]
